# Supplementary material for: Examining the relationship between diarrhea and linear growth in Kenyan HIV-exposed, uninfected infants
Source: PLoS One. 2020 Jul 27;15(7):e0235704. doi: 10.1371/journal.pone.0235704 (PMC7384652; doi:10.1371/journal.pone.0235704)
Supplement: S1 File — (PDF) [file pone.0235704.s002.pdf]

Received: 18 February 2019 | Revised: 5 June 2019 | Accepted: 6 June 2019
DOI: 10.1111/mcn.12861

ORIGINAL ARTICLE

WILEY Maternal & Child Nutrition

Birth size and early pneumonia predict linear growth among HIV-exposed uninfected infants

Emily L. Deichsel[1] | Patricia B. Pavlinac[1] | Barbra A. Richardson[1] | Dorothy Mbori-Ngacha[2] | Judd L. Walson[1,3] | Christine J. McGrath[1] | Carey Farquhar[1] | Rose Bosire[4] | Elizabeth Maleche-Obimbo[5] | Grace C. John-Stewart[1]

[1] Biostatistics, Global Health, Epidemiology, Medicine, University of Washington, Seattle, Washington
[2] United Nations Children's Fund (UNICEF), New York, New York
[3] Child Acute Illness and Nutrition (CHAIN) Network, Nairobi, Kenya
[4] Center for Public Health Research, Kenya Medical Research Institute (KEMRI), Nairobi, Kenya
[5] Department of Paediatrics ands Child Health, University of Nairobi, Nairobi, Kenya

Correspondence
Emily L. Deichsel, Biostatistics, Global Health, Epidemiology, Medicine, University of Washington, Box 359909, 325 Ninth Ave, Seattle, WA 98104.
Email: deichsel@uw.edu

Funding information
Eunice Kennedy Shriver National Institute of Child Health and Human Development of the National Institutes of Health, Grant/Award Numbers: 1F31 HD-089507 and R01 HD-23412

Abstract

Stunting remains a global health priority, particularly in sub-Saharan Africa. Identifying determinants of linear growth in HIV-exposed uninfected (HEU) infants can inform interventions to prevent stunting in this vulnerable population. HIV-infected mothers and their uninfected infants were followed monthly from pregnancy to 12-month post-partum in Nairobi, Kenya. Mixed-effects models estimated the change in length-for-age z-score (LAZ) from birth to 12 months by environmental, maternal, and infant characteristics. Multivariable models included factors univariately associated with LAZ. Among 372 HEU infants, mean LAZ decreased from −0.54 (95% confidence interval [CI] [−0.67, −0.41]) to −1.09 (95% CI [−1.23, −0.96]) between 0 and 12 months. Declines in LAZ were associated with crowding (≥2 persons per room; adjusted difference [AD] in 0–12 month change: −0.46; 95% CI [−0.87, −0.05]), use of a pit latrine versus a flush toilet (AD: −0.29; 95% CI [−0.57, −0.02]), and early infant pneumonia (AD: −1.14; 95% CI [−1.99, −0.29]). Infants with low birthweight (<2,500 g; AD: 1.08; 95% CI [0.40, 1.76]) and birth stunting (AD: 1.11; 95% CI [0.45, 1.78]) experienced improved linear growth. By 12 months of age, 46 infants were stunted, of whom 11 (24%) were stunted at birth. Of the 34 infants stunted at birth with an available 12-month LAZ, 68% were not stunted at 12 months. Some low birthweight and birth-stunted HEU infants had significant linear growth recovery. Early infant pneumonia and household environment predicted poor linear growth and may identify a subgroup of HEU infants for whom to provide growth-promoting interventions.

KEYWORDS
HIV exposed uninfected, infant linear growth, Kenya, low birthweight, pneumonia

1 | INTRODUCTION

Stunting (length-for-age z-score [LAZ] < −2), an indicator of chronic undernutrition, is high in sub-Saharan Africa where approximately 36% of children under five are stunted (Black et al., 2013). Poor linear growth is associated with increased infectious disease morbidity and mortality in childhood (Black et al., 2008; Olofin et al., 2013) and combined with undernutrition is an underlying

© 2019 John Wiley & Sons Ltd

cause in nearly one third of all childhood deaths globally (Black et al., 2013; Pelletier, Frongillo, Schroeder, & Habicht, 1995). Linear growth faltering, an abnormally slow rate of height gain (measured as decline in LAZ), in the first two years of life is associated with cognitive delays, poor school achievement, and reduced adult work capacity (Adair et al., 2013; Cheung & Ashorn, 2010; Grantham-McGregor et al., 2007; Haas, Murdoch, Rivera, & Martorell, 1996; Kuklina, Ramakrishnan, Stein, Barnhart, & Martorell, 2006; Powell, Walker, Himes, Fletcher, & Grantham-McGregor, 1995; Victora et al., 2008).

The rapid expansion of effective strategies to prevent mother-to-child transmission (PMTCT) of HIV resulted in a growing population of HIV-exposed infants who are born uninfected (HEU) and remain HIV uninfected (Barron et al., 2013; Goga, Dinh, & Jackson, 2012; Joint United Nations Programme on HIV/AIDS (UNAIDS), 2016). In 2016 alone, more than one million HEU infants were born (Slogrove et al., 2018). HEU infants are born with lower birthweight and shorter length compared with HIV-unexposed uninfected infants (Bailey, Kamenga, Nsuami, Nieburg, & St Louis, 1999; Evans, Humphrey, Ntozini, & Prendergast, 2016; Kurewa et al., 2009; Marinda et al., 2007; Patel et al., 2010; Sofeu et al., 2014; Taha et al., 1995) and experience high rates of stunting within the first five years (Bailey et al., 1999; Bobat, Coovadia, Moodley, Coutsoudis, & Gouws, 2001; McGrath et al., 2012; Newell, Borja, & Peckham, 2003; Patel et al., 2010).

Linear growth during infancy is influenced by a multitude of factors. There is an increasing evidence that maternal and early infant factors predict linear growth during infancy (Danaei et al., 2016; MAL-ED Network Investigators, 2017a), with up to 20% of stunting at one year of age originating in the fetal period (Black et al., 2013). Despite decades of investigation on the relationship between childhood infections and malnutrition, there remains controversy over the long-term effect of common early life infections, including diarrhoea and pneumonia, on childhood linear growth (Black et al., 2013; Danaei et al., 2016; de Onis & Branca, 2016; MAL-ED Network Investigators, 2017a; Prendergast & Humphrey, 2014; Schlaudecker, Steinhoff, & Moore, 2011; Victora, de Onis, Hallal, Blössner, & Shrimpton, 2010; Webb, Manji, Fawzi, & Villamor, 2009). Further assessment of prenatal and early life risk factors that are associated with linear growth faltering in vulnerable populations is needed to help identify appropriate interventions to optimize child growth.

Few studies have examined risk factors for poor linear growth among HEU children, a population in whom risk factors for linear growth faltering may be unique. HEU children may have immune consequences due to HIV exposure in utero and during breastfeeding and may be more likely to be exposed to infectious diseases as a consequence of living in a household with one or more HIV-infected individuals (Evans, Jones, & Prendergast, 2016; Ruck, Reikie, Marchant, & Kollmann, 2016; Slogrove et al., 2016). We examined prenatal and early infant predictors of linear growth during the first year of life among HEU infants.

Key messages

- Some infants who are born small (low birthweight or short length) experience significant linear growth recovery by the end of the first year of life.
- Early infant pneumonia and household environment may be markers for future infant linear growth faltering.
- Additional research is needed to understand distinct mechanisms that contribute to linear growth decline and recovery.
- Interventions to improve linear growth among vulnerable populations such as HEU infants will require a multifaceted approach.

2 | METHODS

2.1 | Study design

The parent cohort study has been described elsewhere (Gichuhi et al., 2005; E. M. Obimbo et al., 2004; E. M. Obimbo et al., 2009). Briefly, HIV-infected pregnant women living in Nairobi, Kenya between 1999 and 2002 were enrolled at ≥32 weeks of gestation and followed with their infants for 12-month post-partum to assess immunologic mechanisms of protection from breastmilk HIV transmission.

2.2 | Clinical procedures

Risk of HIV-transmission via breastmilk was explained to all women during pregnancy, and they were counselled and supported in their choice to breastfeed or formula feed their infant, including provision of free formula. According to national guidelines during the study period, mothers received short-course zidovudine for PMTCT, and those severely immunocompromised (CD4 count <200 cells/μl) received cotrimoxazole prophylaxis. Neither mothers nor infants received antiretroviral therapy (ART) or prophylaxis during breastfeeding.

Sociodemographic and current and past health information were collected at enrolment with a standardized questionnaire in the study clinic. All participants underwent a comprehensive clinical exam that included anthropometric measurements and blood samples for CD4 count and HIV RNA viral load (VL) tests. Mothers and infants were seen at scheduled clinic visits occurring two weeks post-partum and monthly from birth to 12 months of age. At each clinic study visit, infants underwent a physical examination by study physicians; trained staff measured the infant's weight and recumbent length. Mothers were asked about breastfeeding and their infant's illness in the past month including diarrhoea, assessment for pneumonia, and hospitalizations using standardized questionnaires. Mothers were also encouraged to return to the study clinic if their infant was sick, from which morbidity diagnoses were recorded. To assess infant HIV status, dried blood spots were collected at birth and all subsequent study visits for

HIV DNA detection using polymerase chain reaction for HIV DNA *gag* sequences (Panteleeff et al., 1999).

2.3 | Current analysis

The current analysis included singleton or firstborn twins with documentation of sex, at least one negative HIV polymerase chain reaction confirmatory test and at least two recorded length measurements. Infants were excluded from the analysis if they tested positive for HIV at or before one month of age due to likely perinatal HIV infection. Infants remained in the analysis until the end of follow up (age 12 months) or their last HIV-negative test for those who died, tested HIV polymerase chain reaction positive, or were lost to follow up.

Early life infant morbidity exposures included clinician diagnosis of any diarrhoea or pneumonia using the International Management of Child Illness guidelines or maternal report of hospitalization within the six weeks of life (<42 days).

To reduce potential bias due to missing baseline correlate values, we used Markov chain Monte Carlo procedures to impute missing values assuming and underlying multivariable normal model (m = 10 imputations; Sterne et al., 2009). Missing outcome values were not imputed. All correlates in addition to 12-month HIV status, infant sex, infant mortality, and maternal mortality were included in the imputation models. For comparison, we also conducted a complete case analysis. LAZ was calculated using the WHO Anthro software package based on the 2006 WHO Child Growth Standards (WHO Multicentre Growth Reference Study Group, 2006).

Loess curves were used to plot growth over time. We estimated the average LAZ trajectories by environmental, maternal, and infant characteristics using linear spline mixed-effects models with knot points at four and eight months. Knots were selected based on the distribution of data and visual inspection of cohort growth trends. Random effects for the intercept and slope as well as an autocorrelation structure for residual errors were included to account for within-person correlation in the variance estimation. Models included the correlate of interest, spline terms for infant age, and interaction terms between each correlate and age. The multivariable model included correlates associated with LAZ in univariate models (p value < .05). We tested for collinearity using standard error assessments. Predicted LAZ from the univariate mixed-effects models were used to plot growth profiles for correlates included in the multivariable model. We conducted sensitivity analyses to evaluate the influence of error in length measurements. If the difference in length between two consecutive visits indicated a loss in length in centimetres (<0), the LAZ from the previous visit was carried forward to replace the smaller length measurement and the main analysis was replicated using an edited data set. Additionally, we repeated the main analysis in a subset of infants who had survived and remained HIV-negative until 12 months of age to assess survival bias in the main analysis results.

The parent cohort study and current analysis received ethical approval from the Kenyatta National Hospital Ethics and Research Committee and the University of Washington Institutional Review Board. All analyses were conducted in Stata 14 (StataCorp, College Station, Texas), and p < .05 was considered statistically significant.

3 | RESULTS

3.1 | Study population

Overall, 372 singleton or first twins (six twins) born to HIV-positive women in the parent cohort were included in the present analysis (Figure 1). At enrolment, 52% of participants' homes had a pit latrine; 91% had a shared versus household toilet, and 84% of households were considered crowded (defined as ≥2 people per room). Median maternal age was 25 years (interquartile range [IQR]: 22–28), and 42% of mothers had more than a primary education. At 32 weeks of gestation (enrolment), 13% of mothers were undernourished (mid-upper arm circumference [MUAC] < 23.5); 8% were severely immunocompromised (CD4 count < 200/μl), and 30% had high VL (VL > $\log_{10}$ 4 copies/ml).

Six percent of infants were born with low birthweight (<2,500 g), and 13% were stunted at birth; 15 (4%) infants had both low birthweight and stunting at birth. The majority of infants (75%) received some breastmilk with 29% exclusively breastfed for at least three months. In the first six weeks of life, 3% of infants experienced diarrhoea; 3% were diagnosed with pneumonia, and 7% were hospitalized.

3.2 | Growth patterns

Of the 372 infants, 18 acquired HIV, and 29 died before the end of 12-month follow up and were censored at median 92 (IQR: 30–183) and 90 (IQR: 32–180) days at the last HIV-negative test, respectively. On average, infants experienced a deterioration of LAZ during the first year of life, from −0.54 (95% confidence interval [CI]: [−0.67, −0.41]) at birth to −1.09 (95% CI [−1.23, −0.96]) at 12 month, with a mean change in LAZ of −0.55 (95% CI [−0.71, −0.40]; Figure 2). By one year, 17% (46/269) of children were stunted. Among the 34 infants stunted at birth with available 12-month LAZ, 32% (11) remained stunted at one year,

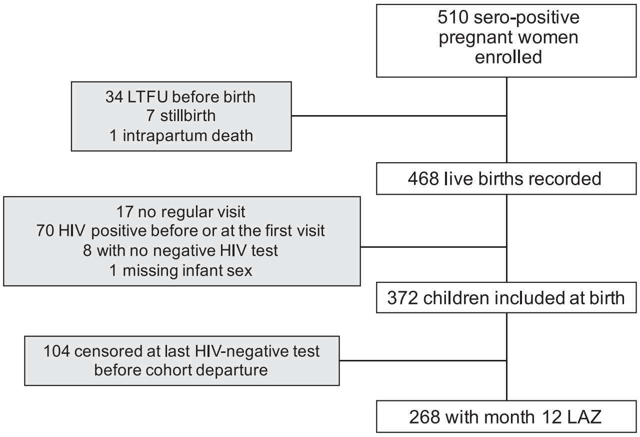

FIGURE 1 Participant flow chart

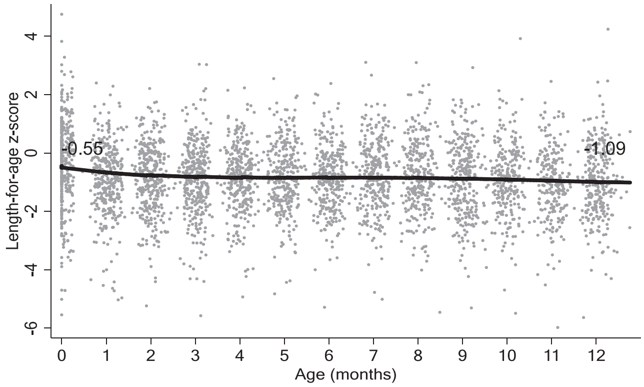

FIGURE 2 Scatter plot change in length-for-age z-score over time by infant age. Loess curve represents modest growth decline over first year

whereas 68% (23) were not stunted at 12 months of age. At 12 months, 46 (17%) infants were stunted, of whom 24% (11) were stunted at birth.

3.3 | Correlates of growth

Socio-economic status and sanitation were associated with change in LAZ from birth to 12 months. Infants in homes with pit latrines (p = .010), shared toilet (p = .032), or crowding (p = .005) experienced a greater deterioration of LAZ (Table 1), despite having similar LAZ at birth for pit latrine and household crowding (Figure 3). Of the household sanitation factors, crowding and use of a pit latrine remained significantly associated with greater declines in LAZ in the multivariable analysis (p = .026 and p = .038, respectively).

Mothers with more than primary education had infants with significantly less deterioration of LAZ (p < .001) compared with mothers with less than primary education, even after adjusting for infant birth size, household toilet type, and early infant pneumonia. Greater maternal height (p = .015) was associated with gains in LAZ, whereas pregnancy mid-upper arm circumference (p = .105) and weight (p = .14) were not associated with LAZ. The significant association between infant growth and maternal height also held after adjustment for low birthweight, stunting at birth, and other correlates. Pregnancy CD4 count (CD4 < 200: p = .939; CD4 200–499: p = .447) and VL (p = .455) were not associated with infant LAZ.

Infants with low birthweight (Figure 3) or stunting at birth had lower LAZ scores for the first year of life relative to their counterparts but experienced gains in linear growth by 12 months (Table 1, both p < .001). The associations remained significant in the multivariable analysis for both variables. In a sensitivity analysis of infants surviving and remaining HIV negative to 12 months of age, low birthweight, and stunting at birth remained significantly associated with gains in linear growth (Table A1). Neither any breastfeeding nor exclusive breastfeeding for more than three months were associated with 0–12 month change in LAZ (p = .099 and p = .438, respectively). Early infant diarrhoea and hospitalization were not associated with changes in LAZ (p = .488 and p = .181, respectively). Early infant pneumonia was associated with declines in LAZ (p = .011), even after adjustment. Infants with early life pneumonia had lower birth LAZ and continued to have lower LAZ and greater LAZ decline from birth to 12 months than infants without early pneumonia (Figure 3).

Results from the sensitivity analyses without imputed data and corrections in infant length measurement error (Tables A2 and A3) were consistent for the univariate analyses. Whereas the main effects in the multivariable analyses were relatively unchanged, household crowding and low birthweight were no longer significantly associated with change in infant LAZ in either sensitivity analysis, and pit latrine was no longer significant in the length measurement error analysis. The results from sensitivity analysis among infants who survived and remained HIV negative differed slightly from the main analysis (Table A1). In the univariate results, shared toilet and maternal height were no longer associated with infant linear growth; additionally, hospitalization was positively associated with infant change in LAZ, among survivors. These discrepancies could be reflections of the unstable independent associations of these correlates or a result of reduced power in the complete case and survival sensitivity analyses.

4 | DISCUSSION

In this cohort of HEU infants, we assessed early life household, maternal, and infant factors associated with linear growth from birth to 12 months of life. HEU infants with low birthweight or stunting at birth experienced some recovery in linear growth and less linear growth decline compared with normal birthweight and nonstunted infants. Most of the HEU infants who were stunted by 12 months of age were not stunted at birth, underscoring the importance of postnatal factors in growth during the first year of life. Early infant pneumonia was independently associated with persistent declines in length throughout the first year of life, whereas early infant diarrhoea and all-cause hospitalization were not. Higher maternal education and maternal height were protected from linear growth faltering, whereas household crowding and lack of access to flush toilets were associated with linear growth declines.

On average, HEU infants experienced a decline of 0.55 LAZ during the first year. The magnitude of this decline is lower than recent cohorts of HEU children in sub-Saharan Africa noting a −1.00 to −1.50 change in z-score in the first year (Islam et al., 2018; MAL-ED Network Investigators, 2017a; Sudfeld et al., 2017; Webb et al., 2009). Mean LAZ at 12 months in this analysis (−0.96) was half that of the Kenya national average at the same age (−1.80; C. G. Victora et al., 2010). This cohort may have had more engagement in health care relative to the general population due to the monthly clinic visits, which included regular counselling on infant nutrition and health, and free infant formula. The prevalence of stunting in the general population increases with age and appears to peak at 24 months of age (Islam et al., 2018; MAL-ED Network Investigators, 2017a; C. G. Victora et al., 2010). Thus, our rates of stunting may be lower because we did not follow children to 24 months during the peak of stunting prevalence.

We noted a strong association between early infant pneumonia and LAZ decline. This relationship persisted after adjusting for socio-

TABLE 1 Change in LAZ scores from 0 to 12 months of 372 Kenyan HEU infants by socio-economic, maternal, and infant factors using linear spline mixed effects models and multiple imputation for missing correlate values

Correlates	*N* (%)[a] or median IQR	LAZ scores Change 0–12 months[b]	Univariate Difference in change 0–12 months (95% CI)[b]	Multivariable Difference in change 0–12 months (95% CI)[c]
Total		−0.55 ± 0.08		
Home environment factors				
Pit latrine	193 (52%)	−0.74 ± 0.10	−0.40 [−0.71, −0.10]*	−0.29 [−0.57, −0.02]*
Flush toilet	179 (48%)	−0.34 ± 0.12	Ref	Ref
Shared toilet	339 (91%)	−0.61 ± 0.08	−0.50 [−0.95, −0.04]*	−0.20 [−0.66, 0.26]
Household toilet	32 (9%)	−0.11 ± 0.22	Ref	Ref
≥2 persons/room in house	314 (84%)	−0.66 ± 0.08	−0.62 [−1.06, −0.18]*	−0.46 [−0.87, −0.05]*
<2 persons/room in house	55 (15%)	−0.03 ± 0.21	Ref	Ref
Maternal factors				
>Primary education	155 (42%)	−0.17 ± 0.12	0.66 [0.35, 0.97]**	0.44 [0.17, 0.71]*
≤Primary education	213 (57%)	−0.83 ± 0.10	Ref	Ref
Primiparous	279 (75%)	−0.60 ± 0.09	−0.18 [−0.52, 0.16]	
Multiparous	90 (24%)	−0.42 ± 0.15	Ref	
Prepartum anthropometry				
Height (10 cm in models)	160 (157–165)	--	0.29 [0.05, 0.53]*	0.22 [0.00, 0.43]*
Weight (10 kg in models)	63 (58–68)	--	0.13 [−0.03, 0.28]	
MUAC < 23.5	50 (13%)	−0.85 ± 0.20	−0.35 [−0.78, 0.07]	
MUAC ≥ 23.5	244 (66%)	−0.50 ± 0.09	Ref	
Prepartum maternal HIV				
CD4 count <200	28 (8%)	−0.59 ± 0.32	0.03 [−0.65, 0.70]	
CD4 count 200–499	189 (51%)	−0.49 ± 0.17	0.13 [−0.20, 0.45]	
CD4 count >500	147 (40%)	−0.62 ± 0.12	Ref	
Viral load ≥4 $\log_{10}$ copies/ml	67 (18%)	−0.59 ± 0.09	−0.15 [−0.53, 0.24]	
Viral load <4 $\log_{10}$ copies/ml	266 (72%)	−0.44 ± 0.17	Ref	
Infant factors				
Birthweight <2,500 g	21 (6%)	1.36 ± 0.39	2.02 [1.23, 2.80]**	1.11 [0.45, 1.78]*
Birthweight ≥2,500 g	343 (92%)	−0.66 ± 0.08	Ref	Ref
Birth LAZ < −2 (stunted)	48 (14%)	0.61 ± 0.20	1.38 [0.95, 1.80]**	1.19 [0.79, 1.59]**
Birth LAZ ≥ −2 (not stunted)	295 (86%)	−0.77 ± 0.08	Ref	Ref
Ever breastfed	279 (75%)	−0.64 ± 0.08	−0.33 [−0.73, 0.06]	
Never breastfed	93 (25%)	−0.31 ± 0.18	Ref	
Exclusively breastfed ≥ first 3 m	107 (29%)	−0.65 ± 0.14	−0.13 [−0.46, 0.20]	
Exclusively breastfed < first 3 m	265 (71%)	−0.52 ± 0.10	Ref	
Infant illness in first month of life				
Diarrhoea	11 (3%)	−0.92 ± 0.53	−0.37 [−1.44, 0.69]	
No Diarrhoea	361 (97%)	−0.54 ± 0.08	Ref	
Pneumonia	13 (3%)	−1.71 ± 0.46	−1.20 [−2.11, −0.28]*	−1.14 [−1.99, −0.29]*
No pneumonia	359 (97%)	−0.51 ± 0.08	Ref	Ref

(Continues)

TABLE 1 (Continued)

Correlates	N (%)[a] or median IQR	LAZ scores Change 0–12 months[b]	Univariate Difference in change 0–12 months (95% CI)[b]	Multivariable Difference in change 0–12 months (95% CI)[c]
Hospitalization	26 (7%)	0.06 ± 0.49	0.66 [−0.31, 1.62]	
No hospitalization	346 (93%)	−0.60 ± 0.08	Ref	

Abbreviations: CI, confidence interval; HEU, HIV-exposed uninfected; IQR, interquartile range; LAZ, length-for-age z-score; MUAC, mid-upper arm circumference.

[a]Percents may not add to 100% due to missing data. Missing values were imputed in models; Missing (n): Shared toilet (1), persons/room (3), maternal education (4), parity (3), maternal height (7), maternal weight (5), maternal MUAC (78), maternal CD4 (8), maternal viral load (39), infant birth stunting (29), and infant birthweight (8).

[b]Estimated from univariate linear spline regression models and multiple imputation for missing correlate values. 95% CIs were calculated using robust variance estimates.

[c]Estimated from multivariable linear spline regression models and multiple imputation for missing correlate values adjusted for pit latrine (vs. flush toilet), shared toilet (vs. household), crowding, maternal education, maternal height, low birthweight, birth-stunting, early infant pneumonia, and age. 95% CIs were calculated using robust variance estimates.

*p value < .05. **p value < .001.

economic factors, maternal height, low birthweight, and birth stunting. Stunting and acute malnutrition are known to increase the risk for acquisition and severity of pneumonia, (Moschovis et al., 2015; Schlaudecker et al., 2011) and although historical studies have shown an association between pneumonia and other respiratory infections and length gains in children age 9–36 months, (Victora, Barros, Kirkwood, & Vaughan, 1990; Walker, Grantham-McGregor, Powell, Himes, & Simeon, 1992) recent studies in older children have not detected an association between respiratory illness and linear growth (MAL-ED Network Investigators, 2017a; Webb et al., 2009). Early infant pneumonia may be more likely to occur in vulnerable children, including HEU infants, with reduced quantity and function of passively acquired maternal antibodies (Abu-Raya, Smolen, Willems, Kollmann, & Marchant, 2016). Pneumonia infection in the first month of life may identify infants with a declining linear growth trajectory influenced either by prenatal factors or their postnatal environment.

We expected that diarrhoea early in life would be associated with linear growth faltering (Checkley, Epstein, Gilman, Cabrera, & Black, 2003; Richard et al., 2013; Scrimshaw, 1970; Venkatesh et al., 2010). However, unlike pneumonia, we did not observe an association between early infant diarrhoea and poor linear growth. Effects of diarrhoea on growth may depend on burden or severity of diarrhoea over the first year of life. Recent evidence suggests that infections with specific enteric pathogens may be responsible for linear growth faltering independent of symptomatic diarrhoea (MAL-ED Network Investigators, 2017b; Rogawski et al., 2018; Schnee et al., 2018).

Infants with low birthweight or stunting at birth had lower LAZ throughout follow up than their counterparts. However, surviving HIV-uninfected infants with low birthweight or short birth length caught up in their LAZ, nearly returning to the levels of their non-low birthweight and birth-stunted counterparts by 12 months of age, consistent with other studies (Webb et al., 2009). Low birthweight has been identified as a mediator of the relationship of HIV exposure and stunting (Sudfeld et al., 2016) and as much as 20% of stunting in the first year of life has been attributed to fetal growth restriction (Black et al., 2013; Christian et al., 2013). We found that almost a third (28%) of infants stunted at 12 months were stunted at birth, suggesting an appreciable impact of fetal growth on long-term stunting. There is some evidence that maternal ART or specific antiretroviral drugs may increase the risk of preterm birth and early stunting in HEU infants (Hofer et al., 2016; Powis et al., 2016; Strydom, Nel, Dhansay, & Van, 2018). As ART continues to be more widely used and the population of HEU children continues to grow, the relative contribution of ART-associated linear growth faltering in the overall prevalence of stunting may be appreciable. With the WHO guidelines recommending lifelong ART among all HIV-infected individuals, including HIV-infected pregnant women, it will be important to identify factors associated with linear growth in the context of longer maternal ART exposure and specific drug regimens.

We found that infants born to mothers with more than primary education or of taller stature experienced less linear growth decline compared with those with less educated and shorter mothers. Maternal education is consistently associated with improved childhood growth in SSA (Omoni et al., 2017; Webb et al., 2009). Among HIV-infected mothers, those with more schooling may have greater knowledge regarding health care seeking, and adherence to medical advice, each of which may help to protect the health of the mother and her infant. Taller maternal stature suggests that linear growth is, in part, informed by genetic potential. This association could also suggest the intergenerational causes of stunting where a mother, stunted since childhood, gives birth to a stunted baby, indicating mother's childhood environment and nutrition contributes to the growth trajectory of her future infant (Black et al., 2013; Martorell & Zongrone, 2012; Prendergast & Humphrey, 2014).

We noted associations between household toilet type and crowding with decline of LAZ. Pit latrines, shared toilets, and household crowding represent contaminated environments and exposure to infectious pathogens that may contribute to growth decline. They

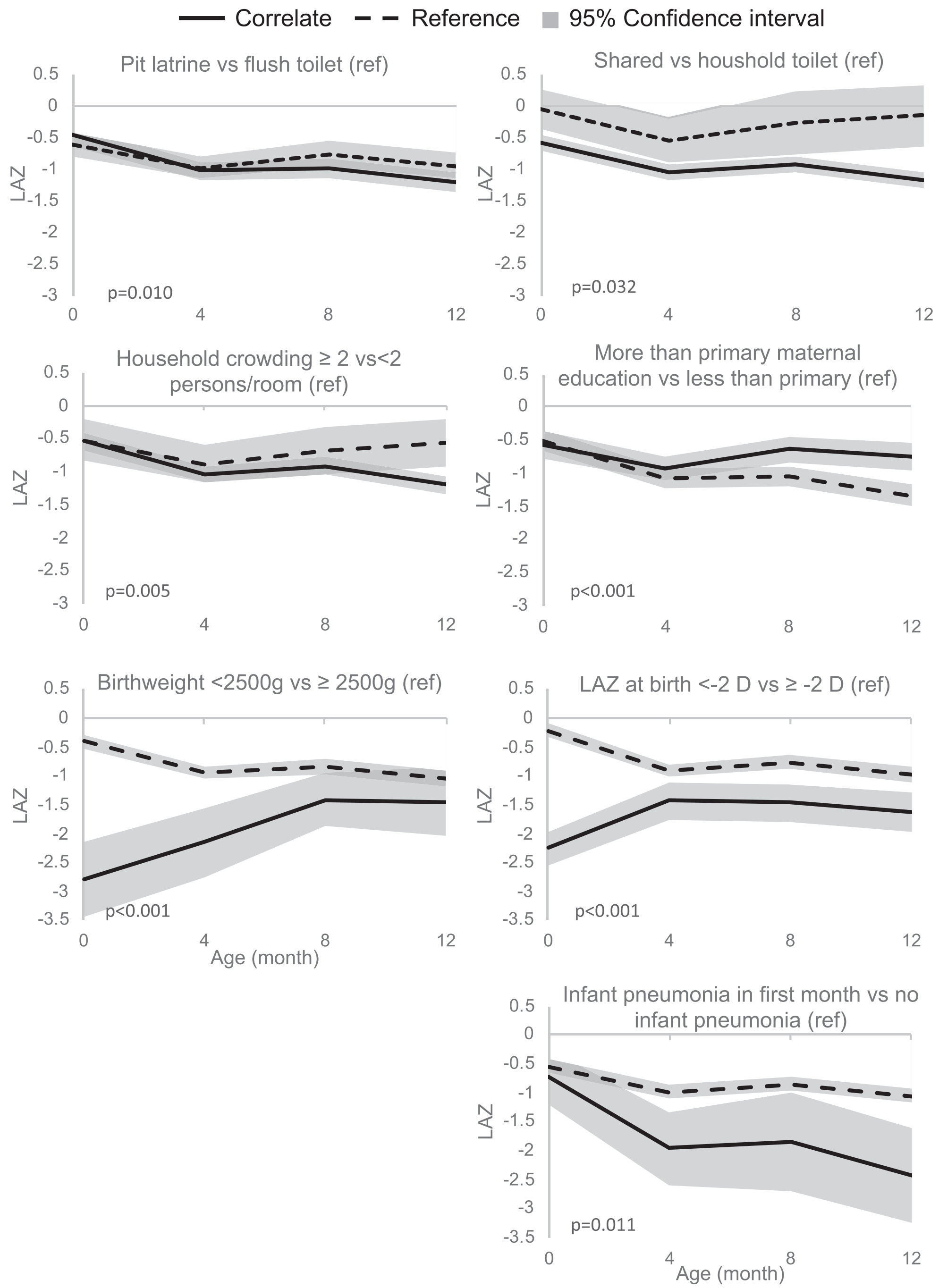

FIGURE 3 Mean predicted length-for-age z-score (LAZ; line) and 95% confidence intervals (grey) over the first year of life for exposure (—) and (– –) reference groups of correlates included in multivariable model. *P* values for difference in change in LAZ from 0 to 12 months from univariate models.

also may represent low socio-economic status, limited resources, and agency to access necessary health care and nutrient rich foods to prevent and treat infections and malnutrition. Because of the complex relationships between these contributing factors, childhood malnutrition and stunting has been difficult to prevent and treat. Targeted interventions to improve household or community sanitation and hygiene have not demonstrated consistent improvement in linear growth (Luby et al., 2018; Null et al., 2018). However, evidence from Brazil suggests broad multisectoral interventions to promote access to and use of health care services as well as household financial stability decreased the prevalence of stunting nationwide (Rasella et al., 2018).

There are limitations to our study. First, the cohort experienced less stunting and had higher 12-month LAZ than anticipated, which decreased statistical power to detect modest associations. Additionally, limited statistical power may explain the lack of significant association of LAZ change with hospitalization and diarrhoea in early infancy. Second, not all infants had length measurements at each of the 12 follow up visits; however, the mixed-effects model addresses interval missingness of the outcome variable by evaluating associations up until

the time of censoring. Third, regression to the mean, cause by measurement error, could partially explain the finding that infants born extremely small tend to have more gains in LAZ. We evaluated the possible effect of regression to the mean by rerunning the low birthweight and birth stunting analyses with and adjustment for the difference between the individuals LAZ value and the group mean LAZ value at enrolment (Barnett, van der Pols, & Dobson, 2005) and found minimal differences in the univariate and adjusted results. Fourth, we did not have an HIV-unexposed comparison group; it is possible that our findings may reflect vulnerable infants in this region in general, not solely HEU. Finally, the cohort received short-course antiretrovirals rather than current Option B+ PMTCT regimens. Although this enabled determination of the influence of maternal immune and viral levels on infant growth, we did not find associations between maternal immune status or VL on growth outcomes suggesting that studies in virally suppressed Option B+ HEU cohorts could yield similar predictors. Nonetheless, the predictors are potentially important in HEU, and perhaps more widely.

In summary, we found although low birthweight infants and infants stunted at birth had linear growth recovery in the first year of life, early infant pneumonia and household sanitation had sustained effects on linear growth trajectory. There are likely distinct mechanisms by which prebirth and postbirth factors contribute to linear growth recovery or decline. Interventions to improve linear growth among HEU infants require a multifaceted approach, including improvement of sanitation, maternal education, and prevention of early life infant infections, to address variety of mechanisms causing linear growth faltering in vulnerable populations.

ACKNOWLEDGMENTS

We would like to thank the women and infants who participated in this study, the research personnel, clinic staff, and data management teams in Nairobi Kenya and Seattle; without whom, this study would not have been possible. We thank the University of Nairobi and Kenyatta National Hospital for providing administrative and physical infrastructure for the study. We thank the University of Washington Global WACh peri-doc mentoring group and Paediatric Infectious Diseases working group, for their comments on the analysis and support during the preparation of this manuscript. We thank Eunice Kennedy Shriver National Institute of Child Health and Human Development of the National Institutes of Health for funding of the parent study as well as this analysis.

CONFLICTS OF INTEREST

The authors declare that they have no conflicts of interest.

CONTRIBUTIONS

ELD, GCJ-S, PBP, JLW, CF, BAR, and CJM conceived an designed the study; GCJ-S, DM-N, EM-O, and RB collected the data; ELD and BAR performed statistical analysis, and ELD, GCJ-S, and PBP drafted the manuscript. All authors contributed to data interpretation and critical revision of the manuscript for intellectual content. All authors read and approved the final manuscript.

ORCID

Emily L. Deichsel https://orcid.org/0000-0002-6172-9458

REFERENCES

Abu-Raya, B., Smolen, K. K., Willems, F., Kollmann, T. R., & Marchant, A. (2016). Transfer of maternal antimicrobial immunity to HIV-exposed uninfected newborns. *Frontiers in Immunology*, *7*(AUG), 1–10. https://doi.org/10.3389/fimmu.2016.00338

Adair, L. S., Fall, C. H. D., Osmond, C., Stein, A. D., Martorell, R., Ramirez-Zea, M., … Victora, C. G. (2013). Associations of linear growth and relative weight gain during early life with adult health and human capital in countries of low and middle income: findings from five birth cohort studies. *Lancet*, *382*(9891), 525–534. https://doi.org/10.1016/S0140

Bailey, R. C., Kamenga, M. C., Nsuami, M. J., Nieburg, P., & St Louis, M. E. (1999). Growth of children according to maternal and child HIV, immunological and disease characteristics: A prospective cohort study in Kinshasa, Democratic Republic of Congo. *International Journal of Epidemiology*, *28*(3), 532–540. https://doi.org/10.1093/ije/28.3.532

Barnett, A. G., van der Pols, J. C., & Dobson, A. J. (2005). Regression to the mean: What it is and how to deal with it. *International Journal of Epidemiology*, *34*(1), 215–220. https://doi.org/10.1093/ije/dyh299

Barron, P., Pillay, Y., Doherty, T., Sherman, G., Jackson, D., Bhardwaj, S., … Goga, A. (2013). Eliminating mother-to-child HIV transmission in South Africa. *Bulletin of the World Health Organization*, *91*(August 2012), 70–74. https://doi.org/10.2471/BLT.12.106807

Black, R. E., Allen, L. H., Bhutta, Z. A., Caulfield, L. E., de Onis, M., Ezzati, M., Mathers C. Rivera, J. (2008). Maternal and child undernutrition: global and regional exposures and health consequences. *The Lancet*, *371*(9608), 243–260. https://doi.org/10.1016/S0140-6736(07)61690-0

Black, R. E., Victora, C. G., Walker, S. P., Bhutta, Z. A., Christian, P., de Onis, M., Ezzati, M., Grantham-McGregor, S., Katz, J., Martorell, R. and Uauy, R. (2013). Maternal and child undernutrition and overweight in low-income and middle-income countries. *The Lancet*, *382*(9890), 427–451. https://doi.org/10.1016/S0140-6736(13)60937-X

Bobat, R., Coovadia, H., Moodley, D., Coutsoudis, A., & Gouws, E. (2001). Growth in early childhood in a cohort of children born to HIV-1-infected women from Durban, South Africa. *Annals of Tropical Paediatrics: International Child Health*, *21*(3), 203–210. https://doi.org/10.1080/02724930120077772

Checkley, W., Epstein, L. D., Gilman, R. H., Cabrera, L., & Black, R. E. (2003). Effects of acute diarrhea on linear growth in Peruvian children. *American Journal of Epidemiology*, *157*(2), 166–175. https://doi.org/10.1093/aje/kwf179

Cheung, Y. B., & Ashorn, P. (2010). Continuation of linear growth failure and its association with cognitive ability are not dependent on initial length-for-age: A longitudinal study from 6 months to 11 years of age. *Acta Paediatrica*, *99*, 1719–1723. https://doi.org/10.1111/j.1651-2227.2009.01593.x

Christian, P., Lee, S. E., Angel, M. D., Adair, L. S., Arifeen, S. E., Ashorn, P., … Hu, G. (2013). Risk of childhood undernutrition related to small-for-gestational age and preterm birth in low- and middle-income countries. *International Journal of Epidemiology*, *42*(5), 1340–1355. https://doi.org/10.1093/ije/dyt109

Danaei, G., Andrews, K. G., Sudfeld, C. R., Mccoy, C., Peet, E., Sania, A., … Fawzi, W. W. (2016). Risk factors for childhood stunting in 137 developing countries: A comparative risk assessment analysis at global, regional, and country levels. *PLoS Medicine*, *13*(11), 1–18. https://doi.org/10.1371/journal.pmed.1002164

de Onis, M., & Branca, F. (2016). Childhood stunting: A global perspective. *Maternal and Child Nutrition*, *12*, 12–26. https://doi.org/10.1111/mcn.12231

Evans, C., Humphrey, J. H., Ntozini, R., & Prendergast, A. J. (2016). HIV-exposed uninfected infants in Zimbabwe: Insights into health outcomes in the pre-antiretroviral therapy era. *Frontiers in Immunology*, *7*(JUN), 1–12. https://doi.org/10.3389/fimmu.2016.00190

Evans, C., Jones, C. E., & Prendergast, A. J. (2016). HIV-exposed, uninfected infants: new global challenges in the era of paediatric HIV elimination. *The Lancet Infectious Diseases*, *16*(6), e92–e107. https://doi.org/10.1016/S1473-3099(16)00055-4

Gichuhi, C., Obimbo, E., Mbori-Ngacha, D., Mwatha, A., Otieno, P., Farquhar, C., … John-Stewart, G. (2005). Predictors of mortality in HIV-1 exposed uninfected post-neonatal infants at the Kenyatta National Hospital, Nairobi. *East African Medical Journal*, *82*(9), 447–451.

Goga, A. E., Dinh, T.-H., & Jackson, D. J. (2012). Evaluation of the effectiveness of the national prevention of mother-to-child transmission (PMTCT) programme measured at six weeks postpartum in South Africa 2010. *South African Medical Research Council, National Department of Health of South Africa, PEPFAR/US Centers for Disease Control and Prevention*. https://doi.org/ISBN: 978-1-920014-87-2 iii

Grantham-McGregor, S., Cheung, Y. B., Cueto, S., Glewwe, P., Richter, L., & Strupp, B. (2007). Developmental potential in the first 5 years for children in developing countries. *Lancet*, *369*, 60–70. https://doi.org/10.1016/S0140-6736(07)60032-4

Haas, J. D., Murdoch, S., Rivera, J., & Martorell, R. (1996). Early nutrition and later physical work capacity. *Nutrition Reviews*, *54*(2), S41–S48.

Hofer, C. B., Keiser, O., Zwahlen, M., Lustosa, C. S., CisneFrota, A. C., de Oliveira, R. H., … Egger, M. (2016). In utero exposure to antiretroviral drugs: Effect on birth weight and growth among HIV-exposed uninfected children in Brazil. *Pediatric Infectious Disease Journal*, *35*(1), 39–46. https://doi.org/10.1016/j.bbi.2015.08.015.Chronic

Islam, M. M., Sanin, K. I., Mahfuz, M., Ahmed, A. M. S., Mondal, D., Haque, R., & Ahmed, T. (2018). Risk factors of stunting among children living in an urban slum of Bangladesh: Findings of a prospective cohort study. *BMC Public Health*, *18*(1), 197. https://doi.org/10.1186/s12889-018-5101-x

Joint United Nations Programme on HIV/AIDS (UNAIDS). (2016). UNAIDS Fact Sheet, (December), 18–25.

Kuklina, E. V., Ramakrishnan, U., Stein, A. D., Barnhart, H. H., & Martorell, R. (2006). Early childhood growth and development in rural Guatemala. *Early Human Development*, *82*(7), 425–433. https://doi.org/10.1016/j.earlhumdev.2005.10.018

Kurewa, E. N., Gumbo, F. Z., Munjoma, M. W., Mapingure, M. P., Chirenje, M. Z., & Rusakaniko, S. (2009). Effect of maternal HIV status on infant mortality: Evidence from a 9-month follow-up of mothers and their infants in Zimbabwe. *Journal of Perinatology*, *30*(2), 88–92. https://doi.org/10.1038/jp.2009.121

Luby, S. P., Rahman, M., Arnold, B. F., Unicomb, L., Ashraf, S., Winch, P. J., Stewart C. P., Begum F., Hussain F., Benjamin-Chung J., Leontsini E., Naser A. M., Parvez S. M., Hubbard A. E., Lin A., Nizame F. A., Jannat K., Ercumen A., Ram P. K., Das K. K., Abedin J., Clasen T. F., Dewey K. G., Fernald L. C., Null C., Ahmed T. Colford, J. M. (2018). Effect of water quality, sanitation, handwashing and nutritional interventions on diarrhoea and child linear growth in rural Bangladesh: A cluster randomized trial. *SUBMITTED. The Lancet Global Health*, *6*(3), e302–e315. https://doi.org/10.1016/S2214-109X(17)30490-4

MAL-ED Network Investigators (2017a). Childhood stunting in relation to the pre- and postnatal environment during the first 2 years of life: The MAL-ED longitudinal birth cohort study. *PLoS Medicine*, *14*(10), 1–21.

MAL-ED Network Investigators (2017b). Relationship between growth and illness, enteropathogens and dietary intakes in the first 2 years of life: Findings from the MAL-ED birth cohort study. *BMJ Global Health*, *2*(4), e000370. https://doi.org/10.1136/bmjgh-2017-000370

Marinda, E., Humphrey, J. H., Iliff, P. J., Mutasa, K., Nathoo, K. J., Piwoz, E. G., … Ward, B. J. (2007). Child mortality according to maternal and infant HIV status in Zimbabwe. *The Pediatric Infectious Disease Journal*, *26*(6), 519–526. https://doi.org/10.1097/01.inf.0000264527.69954.4c

Martorell, R., & Zongrone, A. (2012). Intergenerational influences on child growth and undernutrition. *Paediatric and Perinatal Epidemiology*, *26*(SUPPL. 1), 302–314. https://doi.org/10.1111/j.1365-3016.2012.01298.x

McGrath, C. J., Nduati, R., Richardson, B. A., Kristal, A. R., Mbori-Ngacha, D., Farquhar, C., & John-Stewart, G. C. (2012). The prevalence of stunting is high in HIV-1-exposed uninfected infants in Kenya. *The Journal of Nutrition*, *142*(4), 757–763. https://doi.org/10.3945/jn.111.148874

Moschovis, P. P., Addo-Yobo, E. O. D., Banajeh, S., Chisaka, N., Christiani, D. C., Hayden, D., … Hibberd, P. L. (2015). Stunting is associated with poor outcomes in childhood pneumonia. *Tropical Medicine & International Health*, *20*(10), 1320–1328. https://doi.org/10.1111/tmi.12557

Newell, M.-L., Borja, M. C., & Peckham, C. (2003). Height, weight, and growth in children born to mothers with HIV-1 infection in Europe. *Pediatrics*, *111*(1), e52–e60. https://doi.org/10.1542/peds.111.1.e52

Null, C., Stewart, C. P., Pickering, A. J., Dentz, H. N., Arnold, B. F., Arnold, C. D., … Colford, J. M. (2018). Effects of water quality, sanitation, handwashing, and nutritional interventions on diarrhoea and child growth in rural Bangladesh: A cluster randomised controlled trial. *The Lancet Global Health*, *6*(1718), 30490–30494. https://doi.org/10.1016/S2214-109X(17)30490-4

Obimbo, E. M., Mbori-Ngacha, D. A., Ochieng, J. O., Richardson, B. A., Otieno, P. A., Bosire, R., … John-Stewart, G. C. (2004). Predictors of early mortality in a cohort of human immunodeficiency virus type 1-infected African children. *The Pediatric Infectious Disease Journal*, *23*(6), 536–543. https://doi.org/10.1097/01.inf.0000129692.42964.30

Obimbo, E. M., Wamalwa, D., Richardson, B., Mbori-Ngacha, D., Overbaugh, J., Emery, S., … John-Stewart, G. (2009). Pediatric HIV-1 in Kenya: Patterns and correlates of viral load and association with mortality. *J Acq Immune Defic Syndr*, *51*(2), 209–215. https://doi.org/10.1097/QAI.0b013e31819c16d8

Olofin, I., McDonald, C. M., Ezzati, M., Flaxman, S., Black, R. E., Fawzi, W. W., Caulfield, L. E., … Danaei, G. for the Nutrition Impact Model Study (anthropometry cohort pooling) (2013). Associations of suboptimal growth with all-cause and cause-specific mortality in children under five years: A pooled analysis of ten prospective studies. *PLoS ONE*, *8*(5), e64636. https://doi.org/10.1371/journal.pone.0064636

Omoni, A. O., Ntozini, R., Evans, C., Prendergast, A. J., Moulton, L. H., Christian, P. S., & Humphrey, J. H. (2017). Child growth according to maternal and child HIV status in Zimbabwe. *Pediatric Infectious Disease Journal*, *36*(9), 869–876. https://doi.org/10.1097/INF.0000000000001574

Panteleeff, D. D., John, G., Nduati, R., Mbori-Ngacha, D., Richardson, B., Kreiss, J., & Overbaugh, J. (1999). Rapid method for screening dried blood samples on filter paper for human immunodeficiency virus type 1 DNA. *Journal of Clinical Microbiology*, *37*(2), 350–353.

Patel, D., Bland, R., Coovadia, H., Rollins, N., Coutsoudis, A., & Newell, M.-L. (2010). Breastfeeding, HIV status and weights in South African children: A comparison of HIV-exposed and unexposed children. *Aids*, *24*(3), 437–445. https://doi.org/10.1097/QAD.0b013e3283345f91

Pelletier, D. L., Frongillo, E. A., Schroeder, D. G., & Habicht, J. (1995). The effects of malnutrition on child mortality in developing countries. *Bulletin of World Health Organization*, *73*(5618), 443–448.

Powell, C., Walker, S., Himes, J. H., Fletcher, P., & Grantham-McGregor, S. (1995). Relationships between physical growth, mental development and nutritional supplmentation in stunted children: The Jamacian study. *Acta Paediatrica*, *84*(1), 22–29. https://doi.org/10.1111/j.1651-2227.1995.tb13479.x

Powis, K. M., Smeaton, L., Hughes, M. D., Tumbare, E., Souda, S., Jao, J., … Shapiro, R. L. (2016). In-utero triple antiretroviral exposure associated with decreased growth among HIV-exposed uninfected infants in Botswana. *Aids*, *30*(2), 211–220. https://doi.org/10.1016/j.pmr.2014.05.001.Functional

Prendergast, A. J., & Humphrey, J. H. (2014). The stunting syndrome in developing countries. *Paediatrics and International Child Health*, *34*(4), 250–265. https://doi.org/10.1179/2046905514Y.0000000158

Rasella, D., Basu, S., Hone, T., Paes-Sousa, R., Ocké-Reis, C. O., & Millett, C. (2018). Child morbidity and mortality associated with alternative policy responses to the economic crisis in Brazil: A nationwide microsimulation study. *PLoS Medicine*, *15*(5), e1002570. https://doi.org/10.1371/journal.pmed.1002570

Richard, S. A., Black, R. E., Gilman, R. H., Guerrant, R. L., Kang, G., Lanata, C. F., … Checkley, W. (2013). Diarrhea in early childhood: Short-term association with weight and long-term association with length. *American Journal of Epidemiology*, *178*(7), 1129–1138. https://doi.org/10.1093/aje/kwt094

Rogawski, E. T., Liu, J., Platts-Mills, J. A., Kabir, F., Lertsethtakarn, P., Siguas, M., … Houpt, E. R., and The MAL-ED Network Investigators (2018). Use of quantitative molecular diagnostic methods to investigate the effect of enteropathogen infections on linear growth in children in low-resource settings: Longitudinal analysis of results from the MAL-ED cohort study. *The Lancet. Global Health*, *6*(12), e1319–e1328. https://doi.org/10.1016/S2214-109X(18)30351-6

Ruck, C., Reikie, B. A., Marchant, A., & Kollmann, T. R. (2016). Linking susceptibility to infectious diseases to immune system abnormalities among Hiv-exposed uninfected infants. *Frontiers in Immunology*, *7*(August), 1–12. https://doi.org/10.3389/fimmu.2016.00310

Schlaudecker, E. P., Steinhoff, M. C., & Moore, S. R. (2011). Interactions of diarrhea, pneumonia, and malnutrition in childhood. *Current Opinion in Infectious Diseases*, *24*(5), 496–502. https://doi.org/10.1097/QCO.0b013e328349287d

Schnee, A. E., Haque, R., Taniuchi, M., Uddin, J., Alam, M., Liu, J., … Platts-Mills, J. A. (2018). Idetification of etiology-specific diarrhea associated with linear growth faltering in Bangladeshi infants. *American Journal of Epidemiology*, *303*. https://doi.org/10.1093/aje/kwy106/4996079

Scrimshaw, N. S. (1970). Synergism of malnutrition and infection. *JAMA*, *212*(10), 1685–1692.

Slogrove, A. L., Becquet, R., Chadwick, E. G., Côté, H. C. F., Essajee, S. M., Hazra, R., … Powis, K. M. (2018). Surviving and thriving—Shifting the public health response to HIV-exposed uninfected children: Report of the 3rd HIV-exposed uninfected child workshop. *Frontiers in Pediatrics*, *6*(May), 1–5. https://doi.org/10.3389/fped.2018.00157

Slogrove, A. L., Goetghebuer, T., Cotton, M. F., Singer, J., Bettinger, J. A., & Chougnet, C. A. (2016). Pattern of infectious morbidity in HIV-exposed uninfected infants and children. *Frontiers in Immunology*, *7*(164), 1–8.

Sofeu, C. L., Warszawski, J., Ndongo, F. A., Penda, I. C., Nadiang, S. T., Guemkam, G., … Obedat, S. (2014). Low birth weight in perinatally HIV-exposed uninfected infants: Observations in urban settings in Cameroon. *PLoS ONE*, *9*(4), e93554. https://doi.org/10.1371/journal.pone.0093554

Sterne, J. A. C., White, I. R., Carlin, J. B., Spratt, M., Royston, P., Kenward, M. G., … Carpenter, J. R. (2009). Multiple imputation for missing data in epidemiological and clinical research: Potential and pitfalls. *BMJ (Clinical Research Ed.)*, *338*(July), 157–160. https://doi.org/10.1136/bmj.b2393

Strydom, K., Nel, D. G., Dhansay, M. A., & Van, E. (2018). Paediatrics and International Child Health: The effect of maternal HIV status and treatment duration on body composition of HIV-exposed and HIV-unexposed preterm, very and extremely low- birthweight infants. *Paediatrics and International Child Health*, *9047*(May), 1–12. https://doi.org/10.1080/20469047.2018.1466481

Sudfeld, C. R., Lei, Q., Chinyanga, Y., Tumbare, E., Khan, N., Dapaah-Siakwan, F., … Powis, K. M. (2016). Linear growth faltering among HIV-exposed uninfected children. *Journal of Acquired Immune Deficiency Syndromes*, *73*(April), 1–189. https://doi.org/10.1097/QAI.0000000000001034

Sudfeld, C. R., Manji, K. P., Smith, E. R., Aboud, S., Kisenge, R., Fawzi, W. W., & Duggan, C. P. (2017). Vitamin D deficiency is not associated with growth or the incidence of common morbidities among Tanzanian infants. *Journal of Pediatric Gastroenterology and Nutrition*, *65*(4), 467–474. https://doi.org/10.1097/MPG.0000000000001658

Taha, T. E., Dallabetta, G. A., Canner, J. K., Chiphangwi, J. D., Liomba, G., Hoover, D. R., & Miotti, P. G. (1995). The effect of human-immunodeficiency-virus infection on birth-weight, and infant and child-mortality in urban Malawi. *International Journal of Epidemiology*, *24*(5), 1022–1029. https://doi.org/10.1093/ije/24.5.1022

Venkatesh, K. K., Lurie, M. N., Triche, E. W., De Bruyn, G., Harwell, J. I., McGarvey, S. T., & Gray, G. E. (2010). Growth of infants born to HIV-infected women in South Africa according to maternal and infant characteristics. *Tropical Medicine and International Health*, *15*(11), 1364–1374. https://doi.org/10.1111/j.1365-3156.2010.02634.x

Victora, Cesar G., Adair, L., Fall, C., Hallal, P. C., Martorell, R., Richter, L., & Sachdev, H. S. (2008). Maternal and child undernutrition: Consequences for adult health and human capital. *The Lancet*, *371*(9609), 340–357. https://doi.org/10.1016/S0140-6736(07)61692-4

Victora, C. G., Barros, F., Kirkwood, B. R., & Vaughan, J. P. (1990). Pneumonia, diarrhea, and growth in the first 4 y of life: A study of 5914 urban Brazilian children. *The American Journal of Clinical Nutrition*, *52*, 391–396. https://doi.org/10.1093/ajcn/52.2.391

Victora, C. G., de Onis, M., Hallal, P. C., Blössner, M., & Shrimpton, R. (2010). Worldwide timing of growth faltering: Revisiting implications for interventions. *Pediatrics*, *125*(3), e473–e480. https://doi.org/10.1542/peds.2009-1519

Walker, S. P., Grantham-McGregor, S. M., Powell, C. A., Himes, J. H., & Simeon, D. T. (1992). Morbidity and the growth of stunted and nonstunted children, and the effect of supplementation. *The American Journal of Clinical Nutrition*, *56*(3), 504–510. https://doi.org/10.1093/ajcn/56.3.504

Webb, A. L., Manji, K., Fawzi, W. W., & Villamor, E. (2009). Time-independent maternal and infant factors and time-dependent infant morbidities including HIV infection, contribute to infant growth faltering during the first 2 years of life. *Journal of Tropical Pediatrics*, *55*(2), 83–90. https://doi.org/10.1093/tropej/fmn068

WHO Multicentre Growth Reference Study Group (2006). *WHO child growth standards: Length/height-for-age, weight-for-age, weight-for-length, weight-for-height and body mass index-for-age: Methods and development*. Geneva: World Health Organization.

How to cite this article: Deichsel EL, Pavlinac PB, Richardson BA, et al. Birth size and early pneumonia predict linear growth among HIV-exposed uninfected infants. *Matern Child Nutr*. 2019;e12861. https://doi.org/10.1111/mcn.12861

APPENDIX A

TABLE A1 Correlates of 0- to 12-month change in LAZ scores of 330 Kenyan HEU infants surviving HIV uninfected through 12 months of age using linear spline mixed-effects models and multiple imputation for missing correlate values

Cofactors	*N* (%)[a] or median IQR	Univariate<br>Difference in change 0–12 months (95% CI)[b]	Multivariable model<br>Difference in change 0–12 months (95% CI)[c]
Home environment factors			
Pit latrine	173 (52%)	−0.43 [−0.73, −0.13]*	−0.34 [−0.62, −0.07]*
Flush toilet	157 (48%)	Ref	Ref
Shared toilet	298 (90%)	−0.39 [−0.85, 0.07]	−0.13 [−0.59, 0.34]
Household toilet	31 (9%)	Ref	Ref
≥2 persons/room in house	277 (84%)	−0.63 [−1.07, −0.18]*	−0.54 [−0.95, −0.12]*
<2 persons/room in house	50 (15%)	Ref	Ref
Maternal factors			
>Primary education	140 (42%)	0.56 [0.25, 0.87]**	0.38 [0.10, 0.65]*
≤Primary education	186 (56%)	Ref	Ref
Primiparous	242 (73%)	−0.10 [−0.44, 0.25]	
Multiparous	86 (26%)	Ref	
Prepartum anthropometry			
Height (10 cm in models)	160 (157, 165)	0.23 [−0.01, 0.47]	0.17 [−0.03, 0.36]
Weight (10 kg in models)	63 (58, 68)	0.10 [−0.07, 0.27]	
MUAC < 23.5	40 (12%)	−0.25 [−0.68, 0.19]	
MUAC ≥ 23.5	224 (68%)	Ref	
Prepartum maternal HIV			
CD4 count < 200	21 (6%)	−0.04 [−0.71, 0.63]	
CD4 count 200–499	171 (52%)	0.10 [−0.22, 0.42]	
CD4 count > 500	132 (40%)	Ref	
Viral load ≥ 4 $\log_{10}$ copies/ml	62 (19%)	−0.09 [−0.48, 0.30]	
Viral load < 4 $\log_{10}$ copies/ml	234 (71%)	Ref	
Infant factors			
Birthweight < 2,500 g	15 (5%)	1.94 [1.03, 2.85]**	1.16 [0.38, 1.93]*
Birthweight ≥ 2,500 g	308 (93%)	Ref	Ref
Birth LAZ < −2 (stunted)	42 (13%)	1.29 [0.85, 1.73]**	1.11 [0.71, 1.52]**
Birth LAZ ≥ −2 (not stunted)	260 (79%)	Ref	Ref
Ever breastfed	247 (75%)	−0.46 [−0.84, −0.08]*	
Never breastfed	83 (25%)	Ref	
Exclusively breastfed ≥3 months	94 (28%)	−0.16 [−0.48, 0.17]	
Exclusively breastfed <3 months	236 (72%)	Ref	
Infant illness in first month of life			
Diarrhoea	10 (3%)	−0.37 [−1.43, 0.69]	
No Diarrhoea	320 (97%)	Ref	
Pneumonia	9 (3%)	−0.77 [−1.37, −0.16]*	−0.83 [−1.45, −0.22]*
No pneumonia	321 (97%)	Ref	Ref

(Continues)

TABLE A1 (Continued)

Cofactors	*N* (%)[a] or median IQR	Univariate Difference in change 0–12 months (95% CI)[b]	Multivariable model Difference in change 0–12 months (95% CI)[c]
Hospitalization	20 (6%)	0.92 [0.09, 1.74]*	
No hospitalization	310 (94%)	Ref	

Abbreviations: CI, confidence interval; HEU, HIV-exposed uninfected; IQR, interquartile range; LAZ, length-for-age z-score; MUAC, mid-upper arm circumference.

[a]Percents may not add to 100% due to missing data; missing (n): shared toilet (1), persons/room (3), maternal education (4), parity (2), maternal height (7), maternal weight (5), maternal MUAC (66), maternal CD4 (6), maternal viral load (34), infant birth stunting, (28), and infant birthweight (7).

[b]Estimated from univariate linear spline regression models and multiple imputation for missing correlate values, 95% CIs were calculated using robust variance estimates.

[c]Estimated from multivariable linear spline regression models and multiple imputation for missing correlate values adjusted for pit latrine (vs. flush toilet), shared toilet (vs. household), crowding, maternal education, maternal height, low birthweight, birth stunting, early infant pneumonia, and age, 95% CIs were calculated using robust variance estimates.

$^{*}p$ value < .05. $^{**}p$ value < .001.

TABLE A2 Complete case sensitivity analysis, correlates of 0- to 12-month change in LAZ scores among 372 HEU infants using linear spline mixed-effects models

Cofactors	Complete case analysis: Univariate Difference in change 0–12 months (95% CI)[a]	Complete case analysis: Multivariable model Difference in change 0–12 months (95% CI)[b]
Home environment factors		
Pit latrine	−0.42 [−0.72, −0.11]*	−0.25 [−0.53, 0.04]
Flush toilet	Ref	Ref
Shared toilet	−0.50 [−0.95, −0.04]*	−0.26 [−0.74, 0.22]
Household toilet	Ref	Ref
≥2 persons/room in house	−0.65 [−1.09, −0.21]*	−0.38 [−0.79, 0.03]
<2 persons/room in house	Ref	Ref
Maternal factors		
>Primary education	0.69 [0.38, 0.99]**	0.42 [0.14, 0.71]*
≤Primary education	Ref	Ref
Primiparous	−0.16 [−0.51, 0.19]	
Multiparous	Ref	
Prepartum anthropometry		
Height (10 cm in models)	0.29 [0.06, 0.52]*	0.25 [0.04, 0.47]*
Weight (10 kg in models)	0.13 [−0.04, 0.30]	
MUAC < 23.5	−0.44 [−0.90, 0.03]	
MUAC ≥ 23.5	Ref	
Prepartum maternal HIV		
CD4 count < 200	0.01 [−0.67, 0.68]	
CD4 count 200–499	0.13 [−0.19, 0.45]	
CD4 count > 500	Ref	
Viral load ≥ 4 $\log_{10}$ copies/ml	−0.18 [−0.57, 0.22]	
Viral load < 4 $\log_{10}$ copies/ml	Ref	

(Continues)

TABLE A2 (Continued)

Cofactors	Complete case analysis Univariate Difference in change 0–12 months (95% CI)[a]	Multivariable model Difference in change 0–12 months (95% CI)[b]
Infant factors		
Birthweight < 2,500 g	2.04 [1.25, 2.83]**	0.44 [−0.14, 1.03]
Birthweight ≥ 2,500 g	Ref	Ref
Birth LAZ < −2 (stunted)	1.61 [1.15, 2.07]**	1.41 [0.99, 1.83]**
Birth LAZ ≥ −2 (not stunted)		
Ever breastfed	−0.34 [−0.73, 0.06]	
Never breastfed	Ref	
Exclusively breastfed ≥3 Mon	−0.13 [−0.45, 0.19]	
Exclusively breastfed <3 Mon	Ref	
Infant illness in first month of life		
Diarrhoea	−0.40 [−1.49, 0.69]	
No Diarrhoea	Ref	
Pneumonia	−1.22 [−2.15, −0.28]*	−1.35 [−2.29, −0.41]*
No pneumonia	Ref	Ref
Hospitalization	0.65 [−0.33, 1.62]	
No hospitalization	Ref	

Abbreviations: CI, confidence interval; HEU, HIV-exposed uninfected; LAZ, length-for-age z-score; MUAC, mid-upper arm circumference.

[a]Sample size for complete case univariate models: pit latrine (372), shared toilet (371), persons/room (369), maternal education (368), parity (369), maternal height (365), maternal weight (367), maternal MUAC (294), maternal CD4 (364), maternal viral load (333), infant birthweight (364), birth LAZ (343), ever breastfed (372), exclusively breastfed (372), diarrhoea, pneumonia (372), and hospitalization (372).

[b]From multivariable linear spline models adjusted for pit latrine (vs. flush toilet), shared toilet (vs. household), crowding, maternal education, maternal height, low birthweight, birth-stunting, pneumonia in first month, and age, 95% CIs were calculated using robust variance estimates. Model includes 338 participants.

*p value < .05. **p value < .001.

TABLE A3 Sensitivity analysis for length measurement error, correlates of 0- to 12-month change in LAZ scores using linear spline mixed effects models in an edited dataset imputed for missing correlate values for 372 HEU infants

Correlate	Univariate Difference in change 0–12 months (95% CI)[a]	Multivariable model Difference in change 0–12 months (95% CI)[b]
Home environment factors		
Pit latrine	−0.36 [−0.68, −0.04]*	−0.22 [−0.51, 0.07]
Flush toilet	Ref	Ref
Shared toilet	−0.47 [−0.93, −0.02]*	−0.27 [−0.74, 0.21]
Household toilet	Ref	Ref
≥2 persons/room in house	−0.53 [−0.98, −0.09]*	−0.40 [−0.82, 0.02]
<2 persons/room in house	Ref	Ref
Maternal factors		
>Primary education	0.59 [0.27, 0.92]**	0.39 [0.10, 0.67]*
≤Primary education	Ref	Ref
Primiparous	−0.26 [−0.61, 0.09]	
Multiparous	Ref	
Prepartum anthropometry		
Height (10 cm in models)	0.36 [0.11, 0.61]*	0.30 [0.08, 0.52]*

(Continues)

TABLE A3 (Continued)

Correlate	Univariate<br>Difference in change 0–12 months (95% CI)[a]	Multivariable model<br>Difference in change 0–12 months (95% CI)[b]
Weight (10 kg in models)	0.13 [−0.05, 0.31]	
MUAC < 23.5	−0.28 [−0.69, 0.13]	
MUAC ≥ 23.5	Ref	
Prepartum maternal HIV		
CD4 count < 200	−0.14 [−0.82, 0.53]	
CD4 count 200–499	0.12 [−0.22, 0.46]	
CD4 count > 500	Ref	
Viral load ≥ 4 $\log_{10}$ copies/ml	−0.12 [−0.54, 0.29]	
Viral load < 4 $\log_{10}$ copies/ml	Ref	
Infant factors		
Birthweight < 2,500 g	1.85 [0.95, 2.74]**	0.84 [0.11, 1.57]*
Birthweight ≥ 2,500 g	Ref	Ref
Birth LAZ < −2 (stunted)	1.61 [1.18, 2.03]**	1.49 [1.07, 1.90]**
Birth LAZ ≥ −2 (not stunted)	Ref	Ref
Ever breastfed	−0.26 [−0.68, 0.15]	
Never breastfed	Ref	
Exclusively breastfed ≥3 months	−0.06 [−0.40, 0.27]	
Exclusively breastfed <3 months	Ref	
Infant illness in first month of life		
Diarrhoea	−0.45 [−1.52, 0.63]	
No Diarrhoea	Ref	
Pneumonia	−0.98 [−2.05, 0.10]	−0.90 [−1.91, 0.11]
No pneumonia	Ref	Ref
Hospitalization	0.69 [−0.34, 1.71]	
No hospitalization	Ref	

Note. If the difference in length between two consecutive visits indicate a loss in length in centimetres (<0), the LAZ from the previous visit was carried forward to replace the LAZ of the smaller length measurement, and the main analysis was replicated using an edited data set.

Abbreviations: CI, confidence interval; HEU, HIV-exposed uninfected; LAZ, length-for-age z-score; MUAC, mid-upper arm circumference.

[a]Estaimted from univariate linear spline regression models and multiple imputation for missing correlate values. 95% CI were calculated using robust variance estimates.

[b]Estimated from multivariable linear spline regression models and multiple imputation for missing correlate values adjusted for pit latrine (vs. flush toilet), shared toilet (vs. household), crowding, maternal education, maternal height, low birthweight, birth-stunting, early infant pneumonia, age. 95% CI were calculated using robust variance estimates.

*p value < .05. **p value < .001.
