# Supplementary material for: Examining the relationship between diarrhea and linear growth in Kenyan HIV-exposed, uninfected infants
Source: PLoS One. 2020 Jul 27;15(7):e0235704. doi: 10.1371/journal.pone.0235704 (PMC7384652; doi:10.1371/journal.pone.0235704)
Supplement: Attachment — Submitted filename: Reviewer Comments-Moderate-to-severe diarrhea is associated with short term declines in linear growth.docx [file pone.0235704.s003.docx]

Reviewer’s Comments on the Article titled: “Moderate-to-severe diarrhea is associated with short term declines in linear growth among HIV-exposed, uninfected infants”

**Under Methodology/Principal Findings**,

1. **Lines 9-10**: Any diarrheal was associated with a trend for LAZ decline (adjusted difference [AD]: 0.05, 95% CI: -0.11, 0.01, p=0.096).
2. **Lines10-12**: Linear growth outcomes were not predicted by cumulative episodes of diarrheal or MSD, or timing of diarrheal during infancy. **Under Author Summary-Lines 5-6**: Severity of diarrheal, cumulative burden of episodes, and timing of diarrheal during infancy may influence the relationship between diarrheal and linear growth.
3. **Under Author Summary; Lines 6-8**: ‘Children who are born to women living with HIV, who are HIV exposed but uninfected (HEU) have poorer growth and more infectious illnesses compared to infants born to mothers without HIV infection’
4. **Under statistical analysis, in para 5 lines 9-10**: “…….exclusive breastfeeding, and maternal education (> vs ≤ primary education).”
5. The research questions, motivations and objectives of the study are apparent unclear. There is need for understanding of those three vital elements of any research by the readers.
6. Can the outcomes of this study, given its limited scope to Nairobi be a true reflection or generalization of the Kenyan’ situation on the subject matter?
7. I am concerned with the study title in its lack of capturing smartly, the central goal of the study. Plausible suggestions:
i. Effects of Diarrheal Incidence on the linear growth in children: Evidence from Kenya
ii. Effects of Diarrheal on Linear Growth among HIV-exposed uninfected Infants, in Namibia, Kenya
iii. Examining Relationship between diarrheal and linear growth faltering among HIV-exposed, uninfected infants
8. What are the distinguishing features of this study from the exiting literature in the research bias?
